# Ephrin type-A receptor 2-antisense RNA1/2 promote proliferation and migration of MDA-MB-231 cells through *EPHA2*-dependent Ras signaling pathway mediated by MAPK8/JNK1, MAPK9/JNK2-NFATC2/NFAT1 and JUND

**DOI:** 10.3389/fmolb.2024.1402354

**Published:** 2024-05-24

**Authors:** Tokifumi Odaka, Ryou Sakamoto, Kazuhiro Kumagai, Kazu Okuma, Mikio Nishizawa, Tominori Kimura

**Affiliations:** ^1^ Laboratory of Microbiology and Cell Biology, Department of Pharmacy, College of Pharmaceutical Sciences, Ritsumeikan University, Kusatsu, Japan; ^2^ Department of Microbiology, Faculty of Medicine, Kansai Medical University, Hirakata, Japan; ^3^ Medical Chemistry Laboratory, Department of Biomedical Sciences, College of Life Sciences, Ritsumeikan University, Kusatsu, Japan

**Keywords:** EPHA2-AS1/2, *EPHA2*, MAPK8/JNK1, MAPK9/JNK2, NFATC2/NFAT1, JUND, proliferation, migration

## Abstract

Ephrin type-A receptor 2 (EPHA2) is a receptor tyrosine kinase that is overexpressed in a variety of cancers, including breast cancer. EPHA2 expression may be causally related to tumorigenesis; therefore, it is important to understand how *EPHA2* expression is regulated. We previously reported that EPHA2 antisense RNA (EPHA2-AS), a natural antisense transcript, is an important modulator of EPHA2 mRNA levels and hence production of EPHA2 protein. EPHA2-AS encodes two splice variants, EPHA2-AS1 and EPHA2-AS2. The two variants are constitutively expressed in a concordant manner with EPHA2 mRNA in human breast adenocarcinoma cell lines and in patient samples, with the highest levels detected in the basal-like/triple-negative molecular subtype of breast cancer cells. In this study, we investigated the mechanism of EPHA2-AS1/2 in triple-negative breast cancer using MDA-MB-231 cells. We performed RNA-seq transcriptome analyses of MDA-MB-231 cells treated with AHCC^®^, which suppressed expression of EPHA2-AS1/2 and EPHA2 mRNA, and EPHA2-AS1/2-silenced MDA-MB-231 cells. Bioinformatics analyses identified 545 overlapping differentially expressed genes that were significantly up- or down-regulated by these treatments. Subsequent functional enrichment analyses of the overlapping genes in combination with *in vitro* assays indicated that EPHA2-AS1/2 may promote the proliferation and migration of MDA-MB-231 cells through the *EPHA2*-dependent Ras signaling pathways mediated by MAPK8/JNK1, MAPK9/JNK2-NFATC2/NFAT1 (proliferation and migration) and JUND (migration). These results thus suggest that EPHA2-AS1/2 may represent a potential molecular target for triple-negative breast cancer treatment.

## 1 Introduction

Receptor tyrosine kinases (RTKs) play critical functions in transmitting extracellular signals to the inside of the cell, thereby regulating numerous functional processes required for the development and maintenance of an organism (Kullander and Klein, 2002). The EPH protein family is the largest family of RTKs in the human genome, with 14 EPH receptors (Arora et al., 2023). EPH receptors and the eight ephrin ligands regulate various physiological processes during embryogenesis such as vascular development, tissue boundary formation, cell migration, Axon guidance and synaptic plasticity (reviewed in (Kullander and Klein, 2002; Poliakov et al., 2004; Pasquale, 2005). EPH receptors have been implicated in cellular processes associated with neoplastic progression, including cellular transformation, metastasis and tumor neovascularization (Nakamoto and Bergemann, 2002; Brantley-Sieders et al., 2005).

The expression of EPHA2, a member of the EPH receptor family, has been linked to various malignant tumors. EPHA2 overexpression has been observed in a variety of cancer models, such as primary and transplanted rodent tumors, human tumor xenografts and primary human tumor biopsies, including breast cancer (summarized in (Brantley-Sieders et al., 2008). Experimentally induced overexpression of EPHA2 caused malignant transformation of the non-tumorigenic human breast epithelial cell line, MCF10A (Zelinski et al., 2001). Conversely, siRNA-induced silencing of EPHA2 expression impaired malignant progression of pancreatic and mesothelioma tumor cell lines and ovarian tumors (Duxbury et al., 2004; Landen et al., 2005; Nasreen et al., 2006). Better understanding of the mechanisms that regulate EPHA2 expression may provide insights into the mechanisms by which EPHA2 mediates cancer progression (Macrae et al., 2005).

Triple-negative breast cancer (TNBC), a specific subtype of breast cancer that does not express estrogen receptor (ER), progesterone receptor (PR), or human epidermal growth factor receptor 2 (HER-2), is characterized by high invasiveness, high metastatic potential, proneness to relapse and poor prognosis. Because TNBC tumors lack ER, PR and HER-2 expression, they are not sensitive to endocrine therapy or HER2-targeted treatment, and standardized treatment regimens are still lacking validated, actionable molecular therapeutic targets (Song et al., 2017; Yin et al., 2020).

TNBCs often, though not always, overlap with the basal-like intrinsic molecular subtype (Zardavas et al., 2015). EPHA2 expression is enriched in the basal-like/triple-negative (referred to herein for simplicity as TNBC) molecular subtype of human breast cancer and correlates with poor recurrence-free survival in human TNBC (Song et al., 2017). Loss of EPHA2 function in both human and genetically engineered mouse models of TNBC reduced tumor growth in culture and *in vivo* (Song et al., 2017). Song and colleagues reported EPHA2 as a novel, clinically relevant molecular target in TNBC that could be targeted to impair pro-proliferation pathways that drive malignancy in this breast cancer subtype.

We recently reported that the opposite strand of the *EPHA* locus (1p36.13) is transcribed to yield EPHA2 antisense RNA (EPHA2-AS), a natural antisense transcript, which plays a crucial role in modulating EPHA2 mRNA levels and hence production of EPHA2 protein. EPHA2-AS is a long non-coding RNA with a poly(A) tail that encodes two splice variants (EPHA2-AS1 and EPHA2-AS2). They are constitutively expressed in a concordant manner with EPHA2 mRNA in human breast adenocarcinoma cell lines and in patient samples, with the highest levels detected in the TNBC subtype of MDA-MB-231 cell line (Okuyama et al., 2020).

AHCC^®^ is a proprietary, standardized extract of cultured mycelia of *Lentinula edodes* that is primarily composed of α-glucan components and exhibits immunomodulatory effects on cytokine expressions (Gao et al., 2006; Mach et al., 2008). AHCC^®^ was shown to destabilize IFNA1 mRNA, iNOS mRNA and several other cytokine mRNAs by reducing the expression of the corresponding AS RNAs (Matsui et al., 2011; Kimura et al., 2013), Nishizawa, personal communication). Whether AHCC^®^ influences the expression of EPHA2-AS1/2 and EPHA2 mRNA has not been examined.

In the present study, we examined the roles and potential mechanisms of EPHA2-AS1/2 in TNBC using MDA-MB-231 cells that express high levels of EPHA2-AS1/2 and AHCC^®^. We demonstrated that AHCC^®^ reduced the expression levels of EPHA2-AS1/2 and EPHA2 mRNA, which were associated with the reduction of proliferation and migration of MDA-MB-231 cells. To identify EPHA2-AS1/2 downstream targets, we performed genome-wide transcriptome analyses of AHCC®-treated and EPHA2-AS1/2-silenced MDA-MB-231 cells. Our results revealed that the EPHA2-AS1/2-*EPHA2* axis regulates proliferation and migration of MDA-MB-231 cells, possibly through the Ras signaling pathways mediated by MAPK8/JNK1, MAPK9/JNK2-NFATC2/NFAT1 (proliferation and migration) and JUND (migration).

## 2 Materials and Methods

### 2.1 Cell culture, chemical and antibodies

Human MDA-MB-231 cells (mammary gland epithelial cells from breast adenocarcinoma; ATCC HTB-26) were maintained in RPMI-1640 medium supplemented with 10% heat-inactivated fetal calf serum (FCS) (R10). AHCC^®^ was generously provided by Amino Up Co. Ltd. (Sapporo, Japan). The primary antibodies used were EphA2 (D4A2) XP^®^ rabbit mAb, JNK1 (2C6) mouse mAb, JNK2 (56G8) rabbit mAb, phospho-SAPK/JNK (Thr183/Tyr185) rabbit antibody, NFAT1 (D43B1) XP^®^ rabbit mAb, JunD (D17G2) rabbit mAb, β-Actin (8H10D10) mouse mAb and β-Actin (13E5) rabbit mAb (all from Cell Signaling Technology, Danvers, MA, United States). The secondary antibodies were horseradish peroxidase (HRP)-conjugated affinity purified goat anti-mouse IgG (H + L) antibody (Proteintech Group Inc., Rosemont, IL, United States) and HRP-conjugated affinity purified goat anti-rabbit IgG (H + L) antibody (CSL). All antibodies were used at 1:1000–2000 dilutions for primary antibodies and at 1:2000–5,000 dilutions for secondary antibodies.

### 2.2 Cell transfection and siRNAs

MDA-MB-231 cells were transfected using Lipofectamine 3000 reagent (Thermo Fisher Scientific, Waltham, MA, United States). Briefly, the cells were plated at 4.0×10^5^ cells/well in a 6-well multi-titer plate for 16 h prior to transfection. DNA-lipid complexes were prepared using 1.5 µg sense oligodeoxynucleotide (seODN) or small interfering RNA (siRNA), 3.75 µL of Lipofectamine 3000 reagent and 4 µL of the P3000 reagent (3.75 µL of the Lipofectamine 3000 reagent was diluted in 125 µL of Opti-MEM (Thermo Fisher Scientific)). seODN or control siRNA (1.5 µg) was diluted in 125 µL of Opti-MEM and 4 µL of the P3000 reagent was added to prepare the master mix of DNA. The master mix was added to the diluted Lipofectamine 3000 at a 1:1 ratio and incubated at room temperature for 15 min. The mixture (250 µL) was then dispensed to each well and cells were cultured for 24 h at 37°C.

To silence the expression of EPHA2-AS1/2, unconjugated locked nucleic acid–modified seODNs with a phosphorothioate backbone at both 5′ and 3′ ends (seODN3 and ncseODN; Gene-Design) were used as described previously (Okuyama et al., 2020). siRNAs for NFAT1 (SASI_Hs01_00195473: 5′-CUG​AUG​AGC​GGA​UCC​UUA​A-3′ and SASI_Hs01_00195473_AS: 5′-UUA​AGG​AUC​CGC​UCA​UCA​G-3′), JUND (SASI_Hs01_00200236: 5′-GCA​UCU​CGC​GCC​UGG​AAG​A-3′ and SASI_Hs01_00200236_AS: 5′-UCU​UCC​AGG​CGC​GAG​AUG​C-3′) and universal negative control siRNA (proprietary sequence) were purchased from Sigma-Aldrich Japan (Tokyo, Japan).

### 2.3 Strand-specific reverse-transcription quantitative polymerase chain reaction (RT-qPCR)

RT-qPCR was performed essentially as described previously (Kimura et al., 2013; Kimura et al., 2015; Okuyama et al., 2020). The sample RNA amounts were first normalized to 18S rRNA by ∆∆Ct (Kimura et al., 2013) and the copy numbers of EPHA2-AS1/2 and EPHA2 mRNA were then determined by linear regression analysis. Ten-fold dilutions of a known concentration of *Xba*I- or *Hind*III-digested pEF-EPHA2-AS1 (Okuyama et al., 2020) were assayed in the same run using strand-specific qPCR. The regression lines from each dilution curve were then used to determine the copy numbers of EPHA2-AS1/2 or EphA2 mRNA in each sample (Okuyama et al., 2020). Details of primer sequences and locations within the corresponding genes were previously published (Okuyama et al., 2020). The primer pairs did not generate PCR products in the absence of RT, and PCR products generated from primer-less RT were subtracted, when applicable, from the PCR products obtained from the strand-specific RT-qPCR.

### 2.4 RNA-seq analysis

Total cellular RNA was isolated from AHCC®-treated or EPHA2-AS1/2-silenced MDA-MB-231 cells using Sepasol-RNA I Super G (Nacalai Tesque, Kyoto, Japan). The RNAs were then treated with a TURBO DNA-free DNase I kit (Thermo Fisher Scientific), as described previously (Okuyama et al., 2020). Ribosomal RNAs were removed from the total cellular RNA samples using the Low input RiboMinus™ eukaryote system v2 (Themo Fisher Scientific).

Total cellular RNA samples from three biological replicates were pooled and sequenced on HiSeq (Hokkaido System Science, Sapporo, Japan) using Illumina TruSeq stranded mRNA and total RNA sample preparation kits (Illumina, San Diego, CA, United States) and paired-end sequencing. The samples had more than 40 million pass filter reads with a base call quality of above 95% of bases with Q30 and above. Reads of the samples were trimmed for adapters and low-quality bases using CLC Genomics Workbench v12.0 (Qiagen, Hilden, Germany) before alignment with the reference genome (Human-hg38 or Genome Research Consortium human build 38 (GRCh38)) and assembled by the Workbench, using Ensemble annotations. The average mapping rate of all samples was 91.2%. The unique alignment was above 82%. There were 1.67%–1.72% unmapped reads. The samples had 0.35%–0.59% ribosomal bases. The percent protein coding bases were 90.48%–90.83%. The mapping statistics were calculated using the internal function of CLC Genomics Workbench. The gene expression quantification in raw count format was performed for all samples using CLC Genomics Workbench by the annotation of Human-hg38 reference genome and normalized by the internal standard implemented in the Workbench.

### 2.5 Bioinformatics analysis

We compared the gene expression profiles between AHCC^®^ (1.25 mg/mL)-treated and mock-treated MDA-MB-231 cells and those of seODN3-transfected and ncseODN-transfected MDA-MB-231 cells and calculated the fold change of gene expression using reads per kilobase of exon per million mapped reads (RPKM) data. As was previously used to identify deregulated genes (Jadaliha et al., 2018), we defined significantly differentially expressed (SDE) genes in EPHA2-AS1/2-silenced MDA-MB-231 cells as those with <1/2-fold (i.e., 0.50-fold) or >2.0-fold expression compared with controls; SDE genes in AHCC®-treated MDA-MB-231 cells were defined as those with <0.67-fold or >1/0.67-fold (i.e., 1.49-fold) expression compared with controls. Venn diagram analysis was performed to identify common SDE genes between AHCC®-treated MDA-MB-231 cells and EPHA2-AS1/2-silenced MDA-MB-231 cells.

### 2.6 Functional enrichment analysis

To explore the biological function and processes of genes involved in the EPHA2-AS1/2 signaling pathways, Database for Annotation, Visualization and Integrated Discovery (DAVID) was used to analyze the common SDE genes (Sherman et al., 2022). Using the Gene Ontology (GO) enrichment of DAVID, we compared and classified the common SDE genes to gain insight into their biological functions. The Kyoto Encyclopedia of Genes and Genomes (KEGG) of DAVID allowed analysis of potential pathways of the common SDE genes.

### 2.7 Cell proliferation assay

MDA-MB-231 cells were cultured in growth media supplemented with various concentrations of AHCC^®^ (Kimura et al., 2013). AHCC®-treated cells were harvested at various time points and stained with trypan blue, as described by Shukla and Mishra (Shukla and Mishra, 2018). The number of MDA-MB-231 cells that excluded the dye was counted to create the cell growth curves.

### 2.8 5-Ethynyl-2′-deoxyuridine (EdU) cell proliferation assay

MDA-MB-231 cells were plated in a 24-well tissue culture plate at 6.25×10^5^ cells/well in 250 µL R10 and incubated at 37°C for 16 h. The cells were then incubated with 1x EdU DNA label from the EZClick™ EdU cell proliferation kit (BioVision-ABCAM, Waltham, MA, United States) in R10 at 37°C for 3 h. The cells were washed once with 500 µL phosphate-buffered saline (PBS), trypsinized and collected by centrifugation at 300 *g* for 5 min at 4°C. Cell pellets were resuspended in 150 µL of Fixative Solution and incubated for 15 min at room temperature protected from light. The fixative was removed by centrifugation and the cells were washed twice with 500 µL of 1x EZClick™ Wash Buffer. Cell pellets were resuspended in 100 µL of 1x Permeabilization buffer and incubated for 10 min at RT. The buffer was removed by centrifugation and 100 µL each of 1x EZClick™ Reaction cocktail, including 1x each of Cooper Reagent, EZClick™ Fluorescent Azide and Reducing Agent, was added to resuspend the cell pellets; the mixture was incubated for 30 min at room temperature protected from light. The cells were washed three times with the Wash Buffer; 100 µL of 1x EZClick™ Total DNA Stain was added to the cell pellets and samples were incubated for 20 min at room temperature protected from light. The cells were washed once with PBS. The cell samples were then analyzed by fluorescent activated cell sorter analysis, using a BD FACSCanto II flow cytometer (Franklin Lakes, NJ, United States). The samples were analyzed in FL-2 channel for signals generated by fluorescent EdU-labeled DNA. Data were analyzed using FlowJo software in the FACSCanto II system. At least 5000 cells were measured per sample and the number of EdU-positive cells in the untreated control samples was used to normalize the experimental cell samples.

### 2.9 Wound-healing assay

In preliminary experiments, the concentrations of FCS were titrated by the cell proliferation assay to determine the concentration of FCS at which MDA-MB-231 cells do not proliferate but migrate to close cell-free gaps. For migration assays, MDA-MB-231 cells were then seeded at 6.25×10^5^ cells/well of a 6-well multi-titer plate containing R10 medium. After 16 h incubation at 37°C, the cells were transfected with either seODN3 or siRNAs and incubated for another 24 h at 37°C. A sterile p200 pipette tip was used to create a scratch in the confluent cell monolayer. The wells were washed with PBS and the cells were then cultured with R1 (RPMI-1640 medium supplemented with 1% heat-inactivated FCS). The migration of cells into the wounded areas were imaged using a Leica AF7000 Live Imaging System (Leica Microsystems, Wetzlar, Germany). The number of migrating cells was counted using ImageJ (https://imagej.nih.gov/ij/download.html).

### 2.10 Western blotting assay

Cells were lysed and sonicated in cell lysis buffer [radio immunoprecipitation assay (RIPA)-SDS buffer: 150 mM NaCl, 1.0% NP-40, 0.5% sodium deoxycholate, 0.1% sodium dodecyl sulfate (SDS), 50 mM Tris-HCl pH 7.4], supplemented with complete proteinase inhibitor cocktail (Roche Applied Science, Penzberg, Germany) and phosphatase inhibitor cocktail (Nacalai Tesque, Kyoto, Japan). Homogenized lysates (10–20 µg) and a molecular size marker (Precision Plus Protein™ Kaleidoscope™; BioRad, United States) were separated by 4%–12% SDS-polyacrylamide gel electrophoresis, using Bolt™ Bis-Tris plus mini protein gels (Invitrogen™, Thermo Fisher Scientific) with Bolt™ MES SDS running buffer (Invitrogen™, Thermo Fisher Scientific), followed by electroblotting to polyvinylidene difluoride Immobilon-P membranes (Millipore, Burlington, MA, United States). The membranes were then incubated with the following blocking buffers: Blocking One-P (Nacalai Tesque) for phospho-SAP/JNK antibody, tris-buffered saline with 0.1% Tween20 (TBS-T) supplemented with 5% skim milk (Fujifilm, Tokyo, Japan) for JNK2 (56G8) rabbit monoclonal antibody (mAb) or Blocking One (Nacalai Tesque) for the rest of the primary antibodies. After washing with TBS-T, the blocked membranes were then incubated overnight at 4°C with primary antibodies diluted with Immuno-enhancer A buffer (Fujifilm) or the JNK2 (56G8) mAb diluted with 5% BSA/TBS-T. After another TBS-T wash, the membranes were further incubated with HRP-conjugated second antibody diluted with Immuno-enhancer B buffer (Fujifilm). The blots incubated with the JNK2 (56G8) mAb were incubated with secondary antibody diluted in 5% skim milk/TBS-T. After incubation for 1 h at room temperature, the membranes were washed with TBS-T five times, visualized and analyzed by an Immuno Star Zeta system (Fujifilm) with VILBER FUSION solo S (VILBER BIO IMAGING, Osaka, Japan).

### 2.11 Luciferase reporter assay

The pGL4.32[luc2P/NF-κB-RE/Hygro] vector was purchased from Promega (Madison, WI, United States). AHCC®-treated MDA-MB-231 cells were co-transfected with 1.5 µg of the EPHA2-AS expression vector (pEF-EPHA2-AS1) (Okuyama et al., 2020) or pUC12, 0.3 µg of the pGL4.32[luc2P/NF-κB-RE/Hygro] vector and 0.2 µg of pRSV-*Renilla LUC* vector (Kimura et al., 2015). The cells were harvested at 24 h after transfection and both firefly luciferase and *Renilla* luciferase activities were measured using the Dual-Luciferase Reporter Assay System (Promega) following the manufacturer’s instructions. The luciferase activity was normalized to the *Renilla* luciferase activity.

### 2.12 Statistical analysis

Results in the figures are representative of at least three independent experiments with three or four samples generating similar findings. Differences presented in the figures were analyzed using the Student’s t-test.

## 3 Results

### 3.1 AHCC^®^ reduced the expression levels of EPHA2-AS1/2, EPHA2 mRNA and EPHA2 protein

**FIGURE 1:**
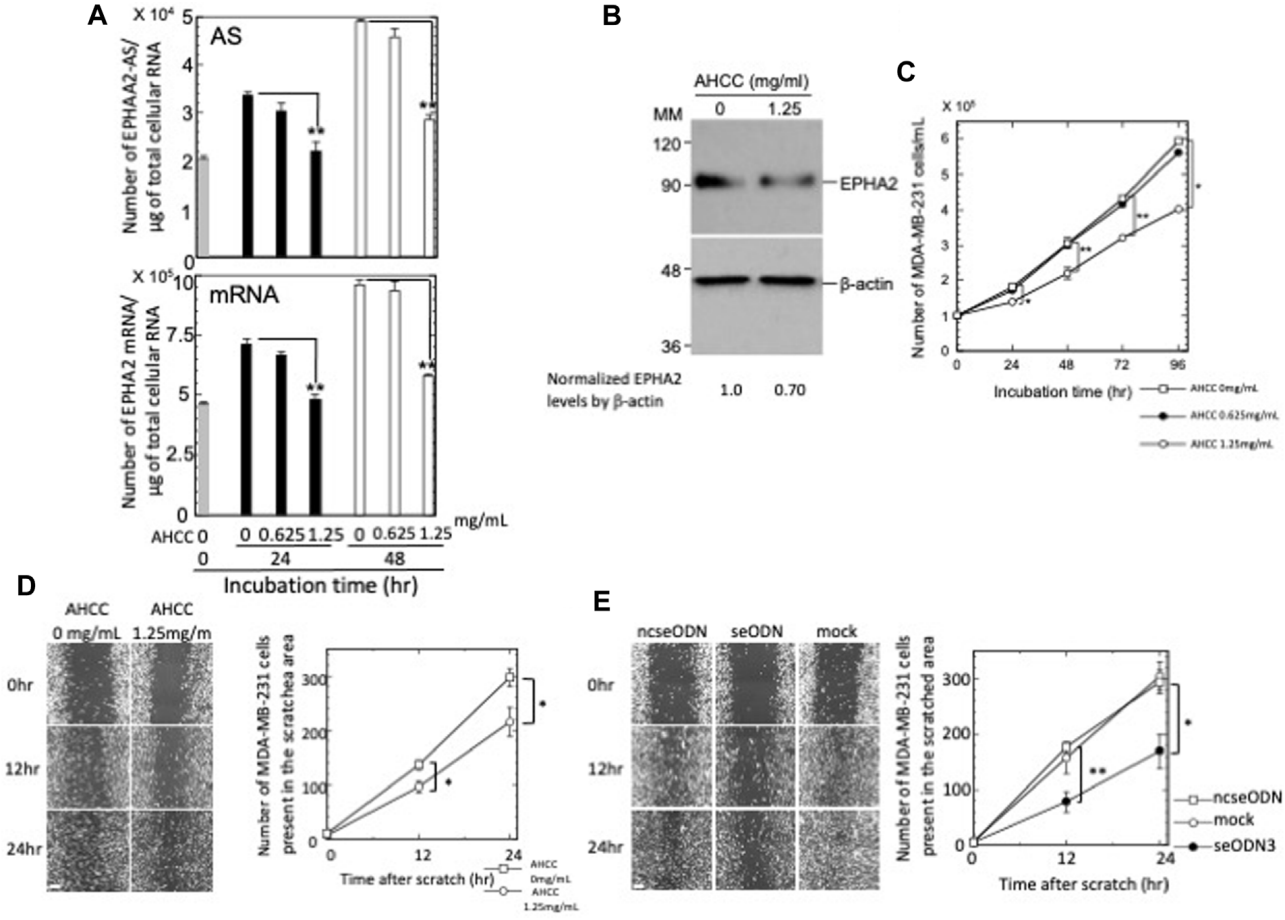
Effects of AHCC^®^ treatment on EPHA2 -AS1/2, EPHA2 mRNA and protein expressions and the proliferation and migration of MDA-MB-231 cells. **(A)** Levels of EPHA2-AS1/2 (top) and EPHA2 mRNA (bottom) in the AHCC®-treated MDA-MB-231 cells were quantified by strand-specific RT-qPCR. Copy numbers of EPHA2-AS1/2 and EPHA2 mRNA were determined as described in the Materials and Methods section. The results are presented as the mean ± s.e.m. of three samples. **(B)** AHCC^®^ (1.25 mg/mL)-treated MDA-MB-231 cells were subjected to western blot analysis after 24 h. Densitometric scanning analysis of immunoblots was performed; the results presented beneath the panel indicates the normalization of EPHA2 to β-actin. MM: molecular size marker (kDa). **(C)** MDA-MB-231 cells were cultured in the presence of various concentrations of AHCC^®^ and the number of live cells were counted after trypan blue staining. **(D)** (*Left*) MDA-MB-231 cells were treated with AHCC^®^ at either 0 or 1.25 mg/mL, scratched with a p200 pipette tip and imaged for 24 h using a microscope equipped with point visiting and a live-cell apparatus. Note that the cells remain in contact during their directed and coordinated movement into the gap. Scale bar = 100 µm. (*right*) The gap areas were imaged at four different sites at each time point and the number of cells that migrated into the areas were counted twice. Values of a representative experiment of three independent experiments are presented as the mean ± s.e.m. of a total eight counts each at the indicated time points. Error bars cannot be seen as they are smaller than the graph symbols. **(E)** (*Left*) MDA-MB-231 cells were transfected with ncseODN or seODN3 and subjected to wound-healing assays. Images are shown from a representative experiment of three independent experiments. Scale bar = 100 µm. (*right*) The number of cells from each transfection group that migrated into the wounded areas were counted. Values are presented as described in the legend **(D)**. *p < 0.05 and **p < 0.01.

Previous studies reported the negative effects of AHCC^®^ on AS RNA expressions (Matsui et al., 2011; Kimura et al., 2013). We thus tested whether this compound might also reduce the levels of EPHA2-AS1/2 expression. Our results showed that AHCC^®^ treatment reduced the levels of EPHA2-AS1/2 and EPHA2 mRNA in a dose-dependent manner (Figure 1A). Exposure of MDA-MB-231 cells to AHCC^®^ at 1.25 mg/mL caused a reduction in the level of EPHA2-AS1/2 to 65.7% (*p* < 0.01) and 58.3% (*p* < 0.01) at 24 h and 48 h, respectively; the same concentration of AHCC^®^ reduced EPHA2 mRNA levels to 67.9% (*p* < 0.01) and 60.3% (*p* < 0.01) at 24 h and 48 h, respectively. Consistent with the mRNA results, western blot analysis showed that AHCC^®^ at 1.25 mg/mL also reduced EPHA2 protein level at 24 h (Figure 1B). These results confirm the effect of AHCC^®^ on inhibiting EPHA2-AS1/2 and EPHA2 mRNA and protein levels.

### 3.2 AHCC^®^ inhibited the proliferation and migration of MDA-MB-231 cells

We next examined the effects of AHCC^®^ on MDA-MB-231 cell activities. Cell proliferation assays showed that AHCC^®^ treatment at 1.25 mg/mL resulted in a time-dependent, statistically significant inhibition of MDA-MB-231 cell proliferation (Figure 1C). The cell growth inhibition rates ranged from 24.1% (*p* < 0.05) at 24 h to 32.1% at 96 h (*p* < 0.05) of treatment.

A recent report (Wei et al., 2020) showed that EPHA2-enriched exosomes from the Panc-1 pancreatic cancer cell line had the ability to transfer cell migration potential to recipient cells. We therefore performed wound-healing assays to examine whether reduction of EPHA2-AS1/2 and EPHA2 mRNA expression levels and hence reduction of EPHA2 protein expression by AHCC®-treatment negatively affected the migration of MDA-MB-231 cells.

We first titrated FCS concentrations for MDA-MB-231 cell migration assays. As shown in Supplementary Figure S1A, significant growth inhibition was observed in cells cultured with FCS concentrations of 0%, 0.3% and 1% compared with the findings in cells cultured with the standard 10% FCS concentration, with comparable inhibition rates among the three concentrations examined. However, in the evaluation of cell migration, cells incubated without FCS (Supplementary Figure S1B) showed significant reduction of migration capabilities throughout the entire assay (*p* < 0.001), whereas cells incubated with 0.3% or 1% FCS showed similar migration capabilities as cells incubated with 10% FCS for the first 12 h and then showed a minor reduction for the next 12 h (Supplementary Figure S1B). These results indicated that 1% FCS significantly inhibited MDA-MB-231 cell proliferation but did not influence migration capabilities, at least for the initial 12 h of incubation. We therefore used 1% FCS for the wound-healing assays.

We next examined the effect of AHCC^®^ at 1.25 mg/mL on MDA-MB-231 cell migration. As shown in Figure 1D, cell migration was repressed by AHCC^®^ at 12 h (note that wild-type cells incubated with 1% FCS showed comparable migration capabilities to that of cells incubated with 10% FCS in the absence of AHCC^®^ at this time point). The inhibition of cell migration was further increased at 24 h. These experiments indicated that AHCC^®^ treatment at 1.25 mg/mL inhibited the proliferation and migration of MDA-MB-231 cells. Consistent with the AHCC^®^ effects on migration of MDA-MB-231 cells, silencing of EPHA2-AS1/2 repressed the migration of cells to 44.3% (*p* < 0.01) and 57.5% (*p* < 0.05) at 12 h and 24 h, respectively, relative to ncseODN-transfected negative control cells (Figure 1E).

### 3.3 Genome-wide transcriptome analyses of AHCC®-treated and EPHA2-AS1/2-silenced MDA-MB-231 cells

To gain insights into the mechanisms of action of EPHA2-AS1/2 in MDA-MB-231 cells, we performed genome-wide transcriptome analyses on MDA-MB-231 cells treated with 1.25 mg/mL of AHCC^®^ and EPHA2-AS1/2-silenced MDA-MB-231 cells and the respective controls. We performed paired-end deep RNA-seq (more than 40 million pass filter reads/sample) on the pooled total cellular RNA samples from three biological replicates of AHCC®-treated and EPHA2-AS1/2-silenced MDA-MB-231 cells and analyzed the expression of 14125 genes in AHCC®-treated (1.25 mg/mL) and 12769 genes in EPHA2-AS1/2-silenced cells (seODN3-transfected cells).

Because the levels of EPHA2 mRNA expression were reduced to 0.67-fold by AHCC^®^ treatment (Figure 1A and Supplementary Table S5), we first identified transcripts that were less than 0.67-fold or more than 1/0.67-fold (i.e., 1.49-fold) deregulated in AHCC®-treated MDA-MB-231 cells. As AHCC^®^ inhibited EPHA2-AS1/2 expression (Figure 1A), we then screened the deregulated transcripts in EPHA2-AS1/2-silenced MDA-MB-231 cells as a positive control that showed less that 0.50-fold or more than 2.0-fold expression (see Materials and Methods section).

**FIGURE 2:**
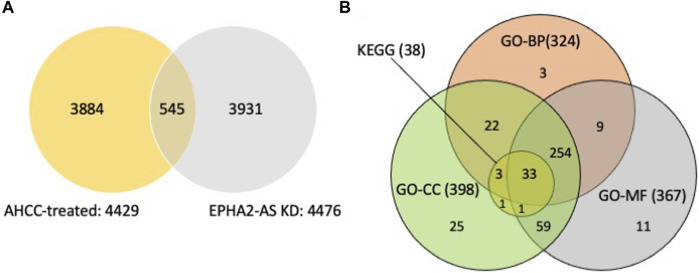
Identification of SDE genes from MDA-MB-231 cells after AHCC^®^ treatment and EPHA2-AS1/2 silencing identified by RNA-seq and functional enrichment analysis of overlapping genes. **(A)** Identification of the SDE genes between AHCC®-treated and EPHA2-AS1/2-silenced MDA-MB-231 cells using a Venn diagram. **(B)** Identification of hub genes from the SDE genes by DAVID. The Venn diagram showed that 33 genes of the 38 genes were enriched in all three GO categories.

A total of 4429 SDE genes in AHCC®-treated MDA-MB-231 cells were identified, including 2732 upregulated genes (more than 1.49-fold change) and 1697 downregulated genes (less than 0.67-fold change) (Figure 2A, Supplementary Table S1). A total of 4476 SDE genes were identified in EPHA2-AS1/2-silenced MDA-MB-231 cells, including 1227 upregulated genes (more than 2-fold change) and 3249 downregulated genes (less than 0.5-fold change). Venn diagram analysis of the SDE genes in the two experimental conditions revealed 545 common genes, including 125 upregulated genes and 420 downregulated genes (Figure 2A).

### 3.4 GO and KEGG analyses of the common SDE genes

We subsequently analyzed the 545 common SDE genes with functional enrichment analysis by GO analysis. As shown in Figure 2B and Supplementary Table S2, 324 genes of the 545 common SDE genes were enriched in 55 biological processes (BP) terms, including ‘regulation of apoptotic signaling pathway,’ ‘gene expression,’ and ‘RNA metabolic process’; the *EPHA2* gene was enriched in 20 BP terms. The results showed that 398 genes of the 545 SDE common genes were enriched in 41 cellular components (CC) terms, including ‘cytoplasm’ and ‘protein complex’; the *EPHA2* gene was enriched in 3 CC terms. Furthermore, 367 genes were enriched in 29 molecular functions (MF) terms, including ‘protein binding’ and ‘phosphatase binding’; the *EPHA2* gene was enriched in 7 MF terms.

We further analyzed the 545 common SDE genes by KEGG enrichment analysis (Figure 2B). We found that 38 genes were significantly involved in 8 KEGG pathways (Supplementary Table S3), and the *EPHA2* gene was involved in the Ras signaling pathway and Axon guidance. Further analysis showed that 33 genes of the 38 genes were enriched in all three GO categories (Figure 2B). We then performed clustergram analysis of these 33 genes (Table 1) and found that mitogen-activated protein kinase 8 (MAPK8) and mitogen-activated protein kinase 9 (MAPK9) were enriched in six of the eight KEGG terms, including the Ras signaling pathway, but not Axon guidance. MAPK14 was enriched in five out of eight KEGG terms but was not enriched in Ras signaling pathway and Axon guidance. Similar clustergram analysis of SDE genes in all the three GO categories with top ten significant terms showed that both MAPK8 and MAPK9 were predicted to participate in all of the GO terms examined (Supplementary Table S4).

**TABLE 1:** Clustergram analysis of the KEGG pathways in which the common SDE genes are predicted to participate.

Common SDE genes	Number of pathways predicted to participate in	Team								
		hsa04622: RIG-I-like receptor signaling pathway		hsa00240: Pyrimidine metabolism	hsa05131: Shigellosis	hsa04014: Ras signaling pathway	hsa04360: Axon guidance	hsa05133: Pertussis	hsa04728: Dopaminergic synapse	hsa05160: Hepatitis C
CFL2	2									
CREB3	1									
CTPS1	1									
EFNA1	2									
EPHA2	2									
EPHB2	1									
GNB1	2									
GNB5	2									
ITGA5	2									
LIMK1	1									
MAPK14	5									
MAPK8	6									
MAPK9	6									
NME1	1									
NME2	1									
NTN1	1									
POLR1B	1									
POLR2J3	1									
POLR2K	1									
PPP2R2A	2									
PRIM1	1									
PSME3	1									
PYCARD	1									
RALB	1									
RHOG	1									
SEMA7A	1									
STAT2	1									
TBK1	3									
TMEM173	1									
TRAF2	1									
U2AF1L4	1									
UPRT	1									
VEGFB	1									

In summary, our genome-wide transcriptome analyses of AHCC®-treated and EPHA2-AS1/2-silenced MDA-MB-231 cells and subsequent functional enrichment analyses of common SDE genes suggested that MAPK8 and MAPK9 might act as hub regulatory genes in the EPHA2-AS1/2-*EPHA2* signaling pathway. Indeed, this finding was further supported by the findings that both Ral guanine nucleotide dissociation stimulator-like 1 (RGL1) and RAS-like proto-oncogene B (RALB) were also recognized as common SDE genes (Supplementary Table S5). RGL1 and RALB have crucial roles downstream of Ras pathway (Zago et al., 2018) and Ras-RALB pathway stimulation results in JNK activation (Essers et al., 2004).

### 3.5 EPHA2-AS1/2-silencing resulted in the reduced expression levels of MAPK8/JNK1, MAPK9/JNK2 and phosphorylated JNK/SAPK (pSAPK/JNK) proteins

**FIGURE 3:**
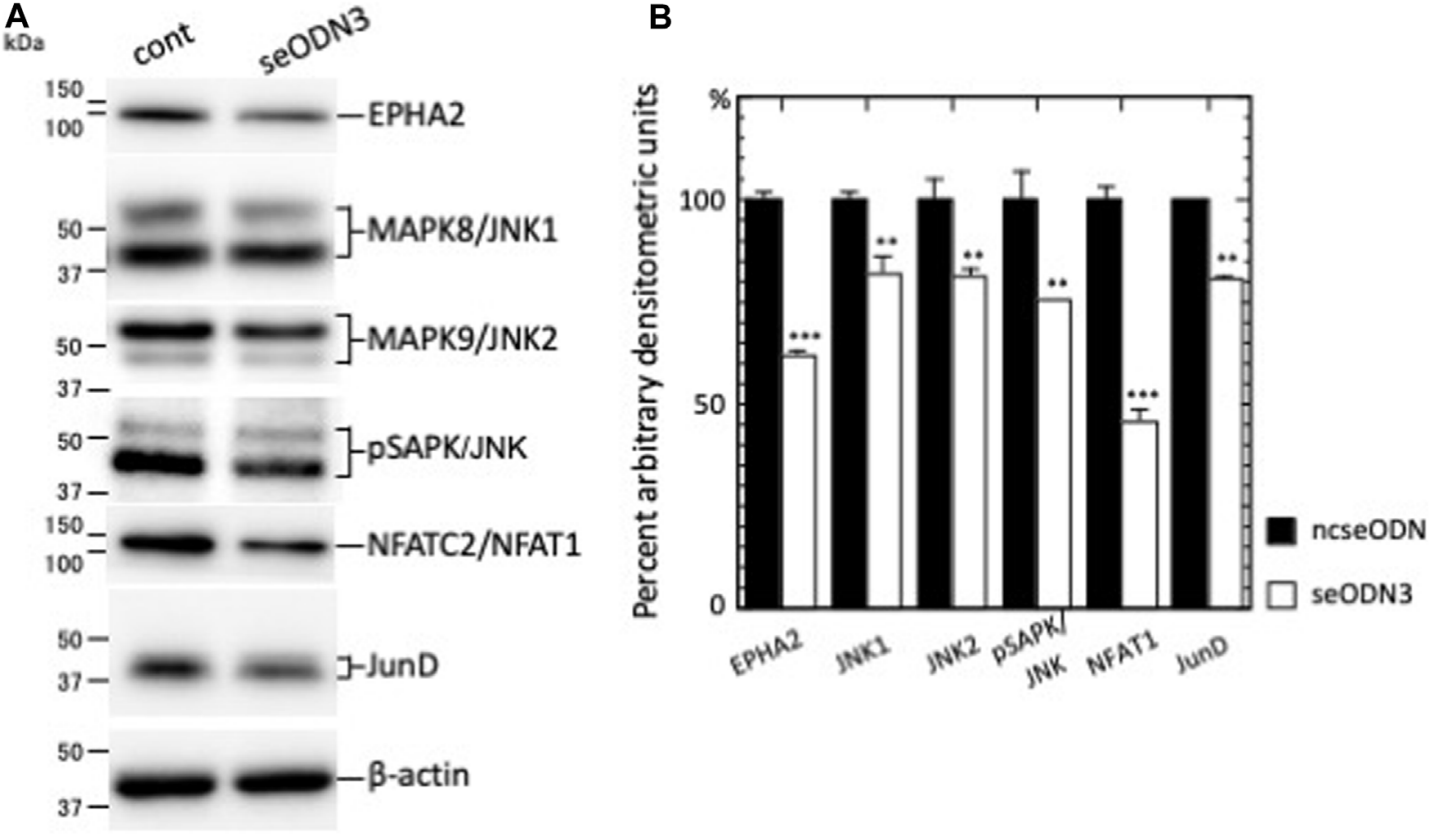
The levels of downstream signaling proteins were reduced upon EPHA2-AS1/2 silencing. **(A)** seODN3 and ncseODN-transfected MDA-MB-231 cells were subjected to western blot analysis. Cells were analyzed 26 h after transfection (except for analysis for JNK2, which was performed at 36 h). MM: molecular size marker (kDa). **(B)** Densitometric scanning analysis was performed and the results were normalized to the densities of β-actin. Relative protein expression levels in EPHA2-AS1/2-silenced cells were calculated from levels in ncseODN-transfected cells. A representative western blot result of three independent experiments is shown. **p < 0.01, and ***p < 0.001.

Our RNA-sequencing results of AHCC®-treated and EPHA2-AS1/2-silenced MDA-MB-231 cells showed that the expression levels of EPHA2 mRNA, MAPK8/JNK1 mRNA and MAPK9/JNK2 mRNA were altered less than 0.67-fold (AHCC®-treated cells) or less than 0.50-fold (EPHA2-AS1/2-silenced cells) relative to negative control cells (Supplementary Table S5). To verify the RNA-seq results, we performed immunoblot analysis of EPHA2-AS1/2-silenced MDA-MB-231 cells. Silencing of EPHA2-AS1/2 reduced the expression of EPHA2, MAPK8/JNK1, MAPK9/JNK2 and pSAPK/JNK to 61.7% (*p* < 0.001) (which was comparable to our previous result (Okuyama et al., 2020)), 82.1% (*p* < 0.01), 81.5% (*p* < 0.01) and 75.8% (*p* < 0.01), respectively, relative to ncseODN-transfected MDA-MB-231 cells (Figures 3A,B). These results suggest that EPHA2-AS1/2-*EPHA2* axis may regulate the MAPK8/JNK1 and MAPK9/JNK2 pathways in MDA-MB-231 TNBC cells.

Both AHCC®-treated cells and EPHA2-AS1/2-silenced cells showed significantly inhibited proliferation and migration (Figure 1). We therefore examined the transcriptome results to identify signaling pathways downstream of MAPK8/JNK1 and MAPK9/JNK2 that may regulate proliferation and migration of MDA-MB-231 cells.

Recruitment of MAPK/JNK into large transcription factor complexes results in MAPK/JNK-mediated phosphorylation of multiple transcription factor substrates (Zeke et al., 2016). We thus next examined several transcription factors and nuclear receptors that are substrates of MAPK/JNK (Supplementary Table S6). Analysis of the RNA-sequencing results revealed that the expression levels of nuclear factor of activated T cells 2 (NFATC2)/NFAT1 mRNA and JunD proto-oncogene, AP-1 transcription factor subunit (JUND) mRNA were altered less than 0.50-fold in the EPHA2-AS1/2-KD MDA-MB-231 cells relative to ncseODN-transfected MDA-MB-231 cells. NFATC2/NFAT1 mRNA expression was also altered less than 0.67-fold in AHCC®-treated MDA-MB-231 cells. Immunoblot analysis showed that silencing of EPHA2-AS1/2 reduced the expression of NFATC2/NFAT1 to 45.7% (*p* < 0.001) and JUND by 80.5% (*p* < 0.01) relative to ncseODN-transfected MDA-MB-231 cells (Figures 3A,B). These results thus suggested that NFATC2/NFAT1 and JUND may participate in the EPHA2-AS1/2-*EPHA2* axis as downstream effectors for MAPK8/JNK1 and MAPK9/JNK2 signaling.

### 3.6 siRNA-mediated knockdown of NFATC2/NFAT1 inhibited the proliferation of MDA-MB-231 cells

**FIGURE 4:**
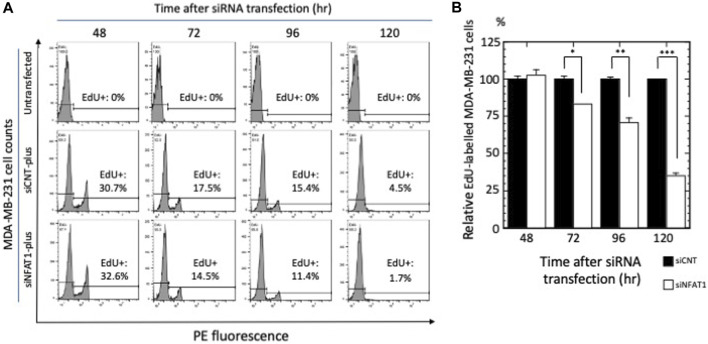
The effects of NFATC2/NFAT1 silencing on MDA-MB-231 cell proliferation. **(A)** Cells were transfected with control siRNA (siCNT) or siRNA against NFATC2/NFAT1 mRNA (siNFAT1) and subjected to 5-EdU cell proliferation assay and flow cytometry. Histograms and percentages of EdU + cells in the total population of MDA-MB-231 cells at the indicated times are shown; a representative experiment of three independent experiments is shown. **(B)** Quantification of the flow cytometric analysis from **(A)**. The number of EdU + cells in siNFAT1-transfected MDA-MB-231 cells were normalized to those in the control siRNA-transfected cells at each time point; data are presented as the ‘relative EdU-labeled MDA-MB-231 cells (%)’ ± s.e.m. of triplicate samples. *p < 0.05, **p < 0.01, and ***p < 0.001.

To examine the effects of NFATC2/NFAT1 and JUND on the cellular phenotypes of MDA-MB-231 cells, we performed EdU cell proliferation assays (Salic and Mitchison, 2008). As shown in Figure 4, silencing of NFATC2/NFAT1 mRNA expression caused a time-dependent reduction of EdU^+^ MDA-MB-231 cells, reflecting a decreased number of proliferating cells to 81.2% (*p* < 0.05) at 72 h, 68.8% at 96 h (*p* < 0.01) and 34.4% at 120 h (*p* < 0.001) compared with the number of negative control siRNA-transfected MDA-MB-231 cells.In contrast, silencing of JUND had no effect on the incorporation of 5-EdU into newly synthesized DNA in MDA-MB-231 cells (Supplementary Figure S2). These results suggested a role for NFATC2/NFAT1 in regulating the proliferation of MDA-MB-231 cells.

### 3.7 Migration of MDA-MB-231 cells was repressed by siRNA-mediated knockdown of NFATC2/NFAT1 and JUND

**FIGURE 5:**
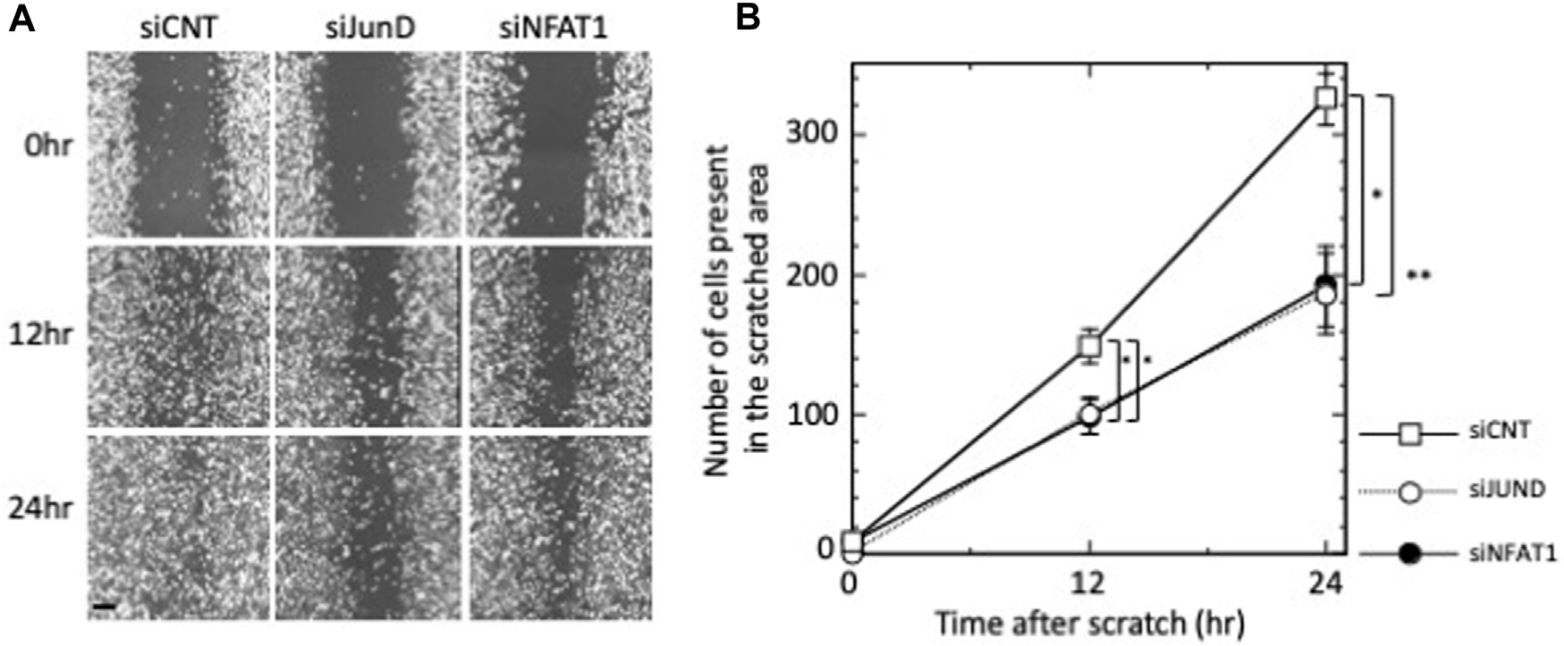
Effects of silencing of JUND, NFATC2/NFAT1 on the migration of MDA-MB-231 cells. **(A)** MDA-MB-231 cells were transfected with control siRNA (siCNT), siRNAs against JUND mRNA (siJUND) or NFATC2/NFAT1 mRNA (siNFAT1) and subjected to wound-healing assay. Images were obtained after 12 h and 24 h. Images are shown from a single representative experiment of three independent experiments. Scale bar = 100 µm. **(B)** The number of cells from each transfection group that migrated into the wounded areas were counted. Values of a representative experiment of three independent experiments are presented as the mean ± s.e.m. of a total eight counts each at the indicated time points.

We next examined the effects of silencing NFATC2/NFAT1 and JUND on the migration of MDA-MB-231 cells. As shown in Figures 5A,B, MDA-MB-231 cell migration was significantly repressed by transfection of siNFAT1 or siJUND at 12 h (*p* < 0.05 for siNFAT1-transfected and siJUND-transfected cells); the number of cells present in the scratched area was reduced to 66.4%, relative to siCNT-transfected negative control cells. The inhibition of MDA-MB-231 cell migration was further increased at 24 h; the number of cells in the scratched area was reduced to 59.0% (*p* < 0.05 for siNFAT1-transfected cells) and 57.2% (*p* < 0.01 for siJUND-transfected cells) relative to siCNT-transfected negative control cells (Figures 5A,B). These results suggested that NFATC2/NFAT1 and JUND may play a role in regulating the migration of MDA-MB-231 cells.

### 3.8 Over-expression of EPHA2-AS reversed the reduction of functional NF-κB expression in AHCC®-treated MDA-MB-231 cells

Our results implied that AHCC^®^ may control the proliferation and migration of MDA-MB-231 cells by suppressing the levels of EPHA2-AS1/2 and EPHA2 mRNA expressions. The common SDE genes identified between AHCC®-treated MDA-MB-231 cells and EPHA2-AS1/2-silenced MDA-MB-231 cells accounted for a portion of SDE genes found in the AHCC®-treated cells, suggesting that AHCC^®^ may exert other effects besides silencing EPHA2-AS1/2. We next performed EPHA2-AS over-expression experiments in AHCC®-treated MDA-MB-231 cells.

As shown in Table 1 and Supplementary Table S5, TANK-binding kinase 1 (TBK1) was identified as a common SDE gene, and this protein is predicted to participate in the Ras signaling pathway. TBK1 interacts with TANK to activate nuclear factor kappa B (NF-κB) (Pomerantz and Baltimore, 1999). NF-κB comprises a family of transcription factors (Oeckinghaus and Ghosh, 2009), and the mRNAs expressions of RELA and RELB proto-oncogene NF-κB subunits (RELA and RELB, respectively) were suppressed upon AHCC®-treatment at 1.25 mg/mL (Supplementary Table S5). We therefore used pGL4.32[*luc2P*/NF-κB-RE/Hygro] vector, which contains copies of an NF-κB response element that drive transcription of the luciferase reporter gene *luc2P*.

As shown in Supplementary Figure S3, treatment of luciferase vector–transfected MDA-MB-231 cells with 1.25 mg/mL of AHCC^®^ significantly reduced the luciferase activity (*p* < 0.01), while co-transfection of an EPHA2-AS expression vector, which increased the levels of EPHA2 mRNA and EPHA2 protein expressions (Okuyama et al., 2020), restored the luciferase activity in the AHCC®-treated MDA-MB-231 cells, indicating that over-expression of EPHA2-AS caused the increase of functional NF-kB expression levels. These results confirmed the functional relevance of silencing EPHA2-AS1/2 in the MDA-MB-231 cells and further reinforced the notion that AHCC^®^ may suppress the levels of EPHA2-AS1/2 and EPHA2 mRNA expressions in regulating the biological phenotypes of MDA-MB-231 cells.

## 4 Discussion

EPHA2 is a direct transcriptional target of the Ras-Raf-MAPK signaling pathway and ligand-stimulated EPHA2 attenuates growth factor-induced activation of this pathway (Macrae et al., 2005). Tumor cells with hyperactive Ras signaling may be suppressed by neighboring cells with ephrin ligand on their cell surface, and an escape from the negative effects of this interaction may be a necessary step in the development of these cancers.

In contrast to the ligand-induced signaling, overexpressed EPHA2 is activated by the interaction with other RTKs in cancer cells, such as epidermal growth factor receptor (EGFR) and HER2/erb-b2 receptor tyrosine kinase 2, leading to amplification of Ras-MAPK signaling and RhoA GTPase activities that promote mammary adenocarcinoma tumorigenesis in a ligand-independent manner (Macrae et al., 2005; Larsen et al., 2007; Brantley-Sieders et al., 2008; Song et al., 2017).

We previously showed that EPHA2-AS1/2 play an important role in modulating EPHA2 mRNA levels and hence production of EPHA2 protein (Okuyama et al., 2020). In this study, we therefore investigated whether EPHA2-AS1/2 may contribute to the malignant activity of TNBC cells and explored the related signaling pathways.

AHCC®is a well-tolerated nutritional supplement that exerts potential antitumor activity (Matsui et al., 2002; Gao et al., 2006; Mach et al., 2008). Our study demonstrates that AHCC^®^ inhibited EPHA2-AS1/2 levels, with subsequent effects on EPHA2 mRNA and protein expression, whose abundant expression has been observed in a variety of cancers, ranging from breast, cervical, ovarian, prostate, esophageal, lung and skin cancers (Xiao et al., 2020) and references cited therein).

Subsequent transcriptomic analyses of MDA-MB-231 cells treated with 1.25 mg/mL of AHCC^®^ and EPHA2-AS1/2-silenced MDA-MB-231 cells and the functional enrichment analyses of common SDE genes suggested that MAPK8/JNK1 and MAPK9/JNK2 of the Ras pathway may be hub regulatory factors in the EPHA2-AS1/2-*EPHA2* signaling axis. This finding was further supported by the findings that RGL1 and RALB, both having crucial roles downstream of the Ras pathway (Zago et al., 2018) but acting upstream of the JNK pathway (Essers et al., 2004), were also among the common SDE genes.

Immunoblotting analyses showed that silencing of EPHA2-AS1/2 resulted in significant reduction of MAPK8/JNK1 and MAPK9/JNK2 protein expression, and decreased pSAPK/JNK protein levels were also observed. These results suggested that EPHA2-AS1/2 axis may regulate MAPK8/JNK1 and MAPK9/JNK2 in Ras signaling in MDA-MB-231 cells.

Both AHCC®-treated cells and EPHA2-AS1/2-silenced cells showed significantly inhibited proliferation and migration. We therefore examined the transcriptome results to identify signaling pathways downstream of MAPK8/JNK1 and MAPK9/JNK2 that may regulate proliferation and migration of MDA-MB-231 cells. Upon analysis of MAPK/JNK substrates, which act on transcription, DNA and chromosome regulation (Zeke et al., 2016), see also Supplementary Table S6), we found NFATC2/NFAT1 expression was significantly reduced by AHCC^®^ treatment and EPHA2-AS1/2 silencing, suggesting that this transcription factor may play a role in the signaling pathway downstream of MAPK8/JNK1 and MAPK9/JNK2 of Ras signaling.

Previous studies reported crucial roles of NFATC2/NFAT1 in promoting breast cancer cell proliferation and metastasis (Qin et al., 2014) and references cited therein). Consistent with these findings, siRNA-mediated gene silencing experiments demonstrated that NFAT1 was involved in the regulation of proliferation and migration of MDA-MB-231 cells. These results implied that MAPK8/JNK1 and MAPK9/JNK2-NFATC2/NFAT1 pathways contribute to the signaling events intrinsic to MDA-MB-231 cells that regulate proliferation and migration.

JUND is also required for TNBC progression (Ibrahim et al., 2018; Dan et al., 2020). Our results showed that EPHA2-AS1/2-silencing reduced the expression of JUND, a MAPK/JNK substrate. siRNA-mediated gene silencing of JUND caused significant reduction of MDA-MB-231 cell migration, consistent with the literature (Ibrahim et al., 2018), but had no impact on cell proliferation. These results thus suggested that JUND may play a role in regulating migration of MDA-MB-231 cells in the signaling pathway downstream of Ras-MAPK8/JNK1 and MAPK9/JNK2.

Song and colleagues previously reported that EPHA2 promotes growth in basal-like/TNBCs through p27/KIP1 inhibition-dependent and c-Myc-dependent mechanisms, which activate cyclin/CDK pathways to promote S phase progression (Song et al., 2017). c-Myc regulates the gene transcription of proteins that sequester p27/KIP1 to enable activation of cyclin E2–associated CDKs for basal-like/TNBC proliferation (Pelengaris et al., 2002). However, our transcriptomic analysis showed that in cells with EPHA2-AS1/2 silencing and AHCC^®^ treatment, cyclin E2 mRNA expression was suppressed, whereas c-Myc mRNA expression was amplified and p27/KIP1 mRNA expression was not elevated but reduced (see Supplementary Table S7). We maintained MDA-MB-231 cells in R10 medium, whereas in the previous study, the cells were starved and then stimulated with serum to study the effects of *EPHA2* silencing for cell cycle progression through S phase (Song et al., 2017). Whether the differences in cell culture conditions may have led to differences in the results is not yet clear.

Our results suggested that AHCC^®^ may reduce the proliferation and migration of MDA-MB-231 cells by suppressing the levels of EPHA2-AS1/2 and EPHA2 mRNA expressions and by reducing the expression of EPHA2, MAPK8/JNK1, MAPK9/JNK2 and NFAT1 protein levels. While the EPHA2-AS over-expression experiment provided a clue to clarify the mechanism of action of AHCC^®^, further experiments will be required to rule out the possibility that AHCC^®^ may influence on other cellular factors that are dependent on or independent of EPHA2.

In the present study, we demonstrate that EPHA2-AS1/2 mediated regulation of EPHA2 mRNA and influenced migration and proliferation of MDA-MB-231 cells, possibly through Ras-MAPK8/JNK1 and MAPK9/JNK2-NFAT1 (for proliferation and migration) and JUND (for migration) pathways. Further studies with other TNBC cell lines and animal models are required to substantiate our findings to establish EPHA2-AS1/2 as novel molecular target for TNBC.

## Data Availability

The data presented in the study are deposited in the DDBJ repository, NCBI Sequence Read Archive (SRA) and EBI Sequence Read Archive (ERA), accession numbers DRA007736 for AHCC®-treated MDA-MB-231 cells and DRA016619 for EPHA2-AS1/2-silenced MDA-MB-231 cells.

## Supplementary material

Supplementary Figure 1 Titration of FCS concentrations for wound healing assays. **(A)** Cell proliferation assay was performed using MDA-MB-231 cells maintained in various concentrations of FCS. Numbers of cells were determined by the trypan blue dye exclusion method. **(B)** MDA-MB-231 cell monolayers were scratched and imaged for 24 h using a microscope equipped with point visiting and a live-cell apparatus. The cells that migrated into the wound area were counted as described in **(A)**. The results at each time point are presented as “number of cells/mL” or “number of cells in the scratched area.” Data are presented as the mean ± s.e.m. of a total of eight counts each at the indicated time point as described in **(A)**. Images are shown from a representative experiment of three independent experiments.

Supplementary Figure 2 The effects of JUND silencing on MDA-MB-231 cell proliferation. **(A)** Cells were transfected with control siRNA (siCNT) or siRNA against JUND mRNA (siJUND) and subjected to 5-EdU cell proliferation assay followed by flow cytometric analyses. Histograms and percentages of EdU+ cells in the total population of MDA-MB-231 cells are shown from a representative experiment of three independent experiments. **(B)** Quantification of the flow cytometric analysis from **(A)**. The number of EdU+ cells in siJUND-transfected MDA-MB-231 cells (open bars) was normalized to those in the control siRNA-transfected cells (closed bars) at each time point and presented as the “relative EdU-labeled MDA-MB-231 cells (%)” ± s.e.m. of triplicate samples.

Supplementary Figure 3 The effect of over-expressed EPHA2-AS1 on NF-kB expression in AHCC®-treated MDA-MB-231 cells. Cells were treated with 1.25 mg/mL of AHCC® and then transfected with the pGL4.32[luc2P/NF-kB-RE/Hygro] vector and the Renilla luciferase plasmid. Luciferase activity was normalized to Renilla luciferase activity, and the results are presented as the mean luciferase activity per μg of protein ± s.e.m. of three samples from a representative experiment of three independent experiments. Over-expression of EPHA2-AS1 restored NF-kB repression observed in AHCC®-treated MDA-MB-231 cells. ***p* < 0.01 and ****p* < 0.001.
