# Supplementary figures and images for: Ephrin type-A receptor 2-antisense RNA1/2 promote proliferation and migration of MDA-MB-231 cells through EPHA2-dependent Ras signaling pathway mediated by MAPK8/JNK1, MAPK9/JNK2-NFATC2/NFAT1 and JUND
Source: Front Mol Biosci. 2024 May 24;11:1402354. doi: 10.3389/fmolb.2024.1402354 (PMC11157115; doi:10.3389/fmolb.2024.1402354)

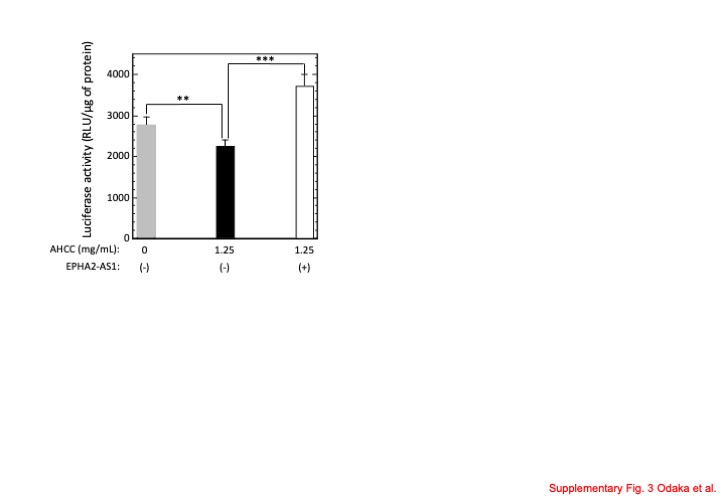

Supplement: Supplementary file 1 [file Image3.jpeg]

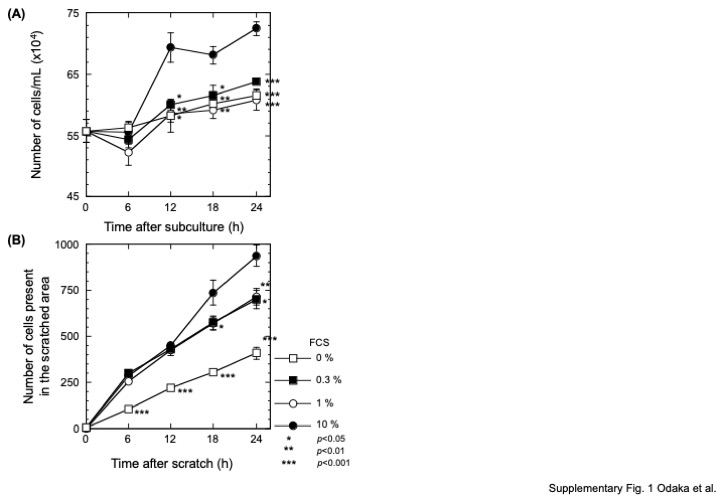

Supplement: Supplementary file 2 [file Image1.jpeg]

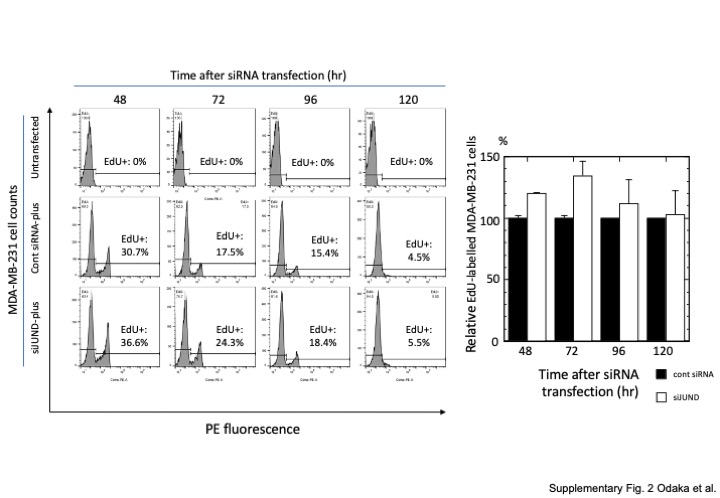

Supplement: Supplementary file 3 [file Image2.jpeg]
